# Changes of Nicotinamide Phosphoribosyltransferase Expressions in Thyroid Glands of Patients with Different Thyroid Pathologies

**DOI:** 10.1155/2018/1316390

**Published:** 2018-02-06

**Authors:** Nadia Sawicka-Gutaj, Mirosław Andrusiewicz, Agata Czarnywojtek, Joanna Waligórska-Stachura, Maciej Biczysko, Jerzy Skrobisz, Jerzy Sowiński, Marek Ruchała

**Affiliations:** ^1^Department of Endocrinology, Metabolism and Internal Medicine, Poznan University of Medical Sciences, Przybyszewskiego St. 49, 60-355 Poznań, Poland; ^2^Department of Cell Biology, Poznan University of Medical Sciences, Rokietnicka St. 5D, 60-806 Poznań, Poland; ^3^Department of Pharmacology, Poznan University of Medical Sciences, Rokietnicka St. 5A, 60-806 Poznań, Poland; ^4^Department of General Surgery and Multiple Trauma and Division of Gastroenterological and Endocrine Surgery, Provincial Hospital, Juraszów St. 7/19, 60-479 Poznań, Poland

## Abstract

**Purpose:**

Our aim was to analyze NAMPT expression in thyroid tissue derived from patients with Graves' disease with (GD) and without (GO) orbitopathy, patients with toxic nodular goiters (TNG) and thyroid cancers (TC), and healthy controls.

**Methods:**

153 thyroid tissue samples of consecutive patients who underwent thyroidectomy were collected. Previous therapy with steroids was an exclusion criterion. We collected clinicopathological data of all subjects and we assessed NAMPT expression using qPCR.

**Results:**

We found the highest NAMPT expression in the thyroids of patients with GO (*n* = 20) and cancers (*n* = 40). Also, there was statistically significant NAMPT overexpression in patients with TNG (*n* = 30). Relatively low NAMPT expression was found in GD patients (*n* = 21) and in the control group (*n* = 39). In one-way ANCOVA, we confirmed that NAMPT expression differs between subgroups and that it is not influenced by age, BMI, or sex of patients.

**Conclusions:**

Reported alteration of NAMPT expression might suggest its involvement in thyroid pathologies. Observed NAMPT overexpression in patients with GO and its relatively low levels in thyroids of patients with GD without eye changes do not confirm causal relationship between NAMPT level and orbitopathy, but this needs further investigation.

## 1. Introduction

Nicotinamide phosphoribosyltransferase (NAMPT), also known as visfatin or pre-B cell colony-enhancing factor (PBEF), is a protein with complex properties [1]. It has enzymatic activity involved in the NAD cellular process. NAMPT has also been identified as a cytokine-like factor with increased concentration in acute and chronic inflammation [2]. Furthermore, changes of NAMPT/visfatin concentrations and expressions have been investigated in autoimmune diseases [3]. Finally, alteration of NAMPT/visfatin/PBEF levels has been observed in diabetes and obesity [4, 5].

NAMPT has also antiapoptotic properties, and its overexpression was observed in many cancers, including thyroid cancers [6, 7]. We have previously found that* NAMPT* expression correlated with thyroid cancer stage and lymph node invasion [6]. Recently, several NAMPT inhibitors have been synthesized, and some of them were tested in clinical trials among patients with cancers that are nonresponsive to conventional therapy [8, 9].

We have previously reported* NAMPT* overexpression in leukocytes of Graves' orbitopathy patients and its correlation with TSH receptor autoantibodies (TRAb) levels [10]. At the same time, we have observed increased NAMPT/visfatin/PBEF serum concentration in patients with Graves' disease with and without orbitopathy. Since NAMPT/visfatin/PBEF is elevated in several autoimmune diseases (e.g., rheumatoid arthritis, inflammatory bowel disease, psoriasis, and systemic lupus erythematosus), we hypothesized that it might be potentially involved in the inflammatory cascade in Graves' orbitopathy. We suggested that upregulation of NAMPT in leukocytes might reflect its potential immunological properties in patients with Graves' orbitopathy. In another study, we have shown that NAMPT/visfatin/PBEF serum concentration in hypothyroid patients is influenced by both free thyroid hormones and anti-thyroperoxidase antibodies [11]. In view of these results, we aimed to analyze* NAMPT* expression in thyroid tissue derived from patients with Graves' disease with and without orbitopathy, patients with toxic nodular goiters and thyroid cancers, and healthy controls.

## 2. Materials and Methods

### 2.1. Tissue Samples

From 2013 to 2016, 153 thyroid tissue samples of consecutive patients who underwent thyroidectomy were collected. Three patients with Graves' orbitopathy who had been treated with systemic steroids before operation were excluded. All patients were clinically and biochemically euthyroid at the time of surgery. There were 41 patients with Graves' disease (20 patients with orbitopathy [GO] and 21 patients without orbitopathy [GD]), 30 patients with toxic nodular goiters (TNG), and 40 cases of thyroid cancers (29 of papillary thyroid cancer, 5 of medullary thyroid cancer, 4 of undifferentiated thyroid cancer, and 2 of follicular thyroid cancer), which were previously described [6]. 39 cases of healthy thyroid tissue derived from healthy regions of removed nodular goiters were controls.

Resected thyroid tissues were immediately submerged in RNA protective medium (RNA Stabilizer, Novazym, Poznan, Poland) and stored until RNA isolation at −80°C.

The study was approved by the Ethical Committee of Poznan University of Medical Sciences. Informed written consent was given by all patients.

### 2.2. RNA Isolation, cDNA Synthesis, and Quantitative PCR

#### 2.2.1. Ribonucleic Acid Isolation

Tissue specimens were pulverized, and standard 3-zone monophasic solution (Novazym, Poznan, Poland) was used for ribonucleic acid isolation according to the manufacturer's protocol. After the isolation, RNA was treated with DNase I (Zymo Research, Irvine, CA, USA) to prevent DNA contamination of RNA. This was followed by a second 3-zone isolation step. Extracted ribonucleic acid was assessed spectrometrically for quantity and purity and electrophoretically verified in denaturing conditions for its integrity.

#### 2.2.2. Reverse Transcription cDNA Synthesis

The reverse transcription reactions were made according to the Transcriptor Reverse Transcriptase manufacturer's protocol (Roche Molecular Diagnostics, Pleasanton, CA, USA). The 20 *μ*l reaction mixture was prepared for 1 *μ*g of total cellular RNA treated with DNase I. After denaturation of RNA secondary structures in the presence of water and oligo(d)T_10_ primer, the 10 U/*μ*l reverse transcriptase, 1x buffer, 10 U/*μ*l RNasin (RNase inhibitor), and 1 pm/*μ*l of each of the deoxynucleotide triphosphates were added and incubated at 55°C for 30 min followed by an enzyme denaturation. The synthesized cDNA was subsequently used in quantitative PCR reactions.

#### 2.2.3. Quantitative Polymerase Chain Reaction

**Figure 1:**
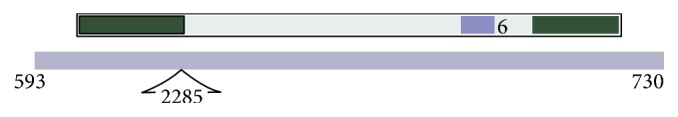
Position of the primers and hydrolysis probe #6 according to the GenBank AC# NM_005746.2 sequence of NAMPT. The intron length at the sense primer position in length of 2285 bp as indicated.

To obtain the specific qPCR product, the TaqMan® hydrolysis probe #6 (Cat. number 04685032001) (Roche) derived from Roche Universal ProbeLibrary and* NAMPT *(GenBank AC#: NM_005746.2) primers generating 118 bp amplicon in length (sense and antisense sequences: 5′-aagggatggaactacattcttgag-3′ and 5′-ctgtgttttccaccgtgaag-3′, resp., Genomed, Warsaw, Poland) were used. The sense primer was designed at the splice junction (Figure 1), preventing genomic DNA product generation.

Human ready to use* HPRT* Gene Assay (Cat. number 05046157001; Roche) served as a reference/normalization reaction. qPCR reactions were performed in a total reaction volume of 20 *μ*l in the LightCycler® 2.0 carousel based thermal cycler system (Roche). All reactions were made in triplicates with the hot start 1x LightCycler® TaqMan® Master Mix (Roche). The hydrolysis probe and primers concentrations for the gene of interest (GOI =* NAMPT*) were set to 200 nM (in the case of the reference gene, a standard 100 nM probe was used). The standard thermal profile for TaqMan® hydrolysis probes was used. In each reaction, no template control was applied. Reaction efficiencies for GOI and reference were calculated by comparing raw data with the genes' corresponding standard curves (generated from qPCR reactions with serial decimal dilutions of pooled cDNA template). The average threshold values, Ct (compared to the standard curves), were normalized to the reference gene acquisition data. The obtained data were shown as derivative of the concentration ratios (Cr). This Cr data was used for statistical analysis.

### 2.3. Statistical Analysis

All calculations were performed with MedCalc statistical software version 16.8 (MedCalc Software Bvba, Ostend, Belgium; https://www.medcalc.org; 2016). A *p* value less than 0.05 was considered to be statistically significant. Normality within subgroups was analyzed by the D'Agostino-Pearson test.* NAMPT* expression was compared between five groups (thyroid glands of patients with Graves' disease with and without orbitopathy, toxic nodular goiters, healthy tissues, and thyroid cancers) by the Kruskal-Wallis test. If the Kruskal-Wallis test was positive (*p* less than the selected significance level), then a test for pairwise comparison of subgroups according to Conover (1999) was performed. One-way analysis of variance was used to compare patients' ages between all groups. Chi-square test was used to compare discrete variables. A one-way ANCOVA was conducted to determine a statistically significant difference of NAMPT expressions between subgroups controlling for age, sex, and BMI. Before comparison, nonnormally distributed values of* NAMPT* expressions were logarithmically transformed toward normality.

## 3. Results

Comparison of age, gender, and BMI between all groups is provided in Table 1. Patients with GO and GD were significantly younger than patients with thyroid cancers; GO patients were also younger than subjects with TNG and healthy controls. There were more males in a subgroup of patients with thyroid cancer as compared to the rest of studied patients. All groups did not differ according to patients' BMI.

All patients with Graves' orbitopathy had active (Clinical Activity Score ≥ 3) and moderate-to-severe GO. Mean time of duration of eye changes reported by patients was 7 ± 2 months. Before surgery, all of GD, GO, and TNG patients had been treated with antithyroid medications due to hyperthyroidism.

**Figure 2:**
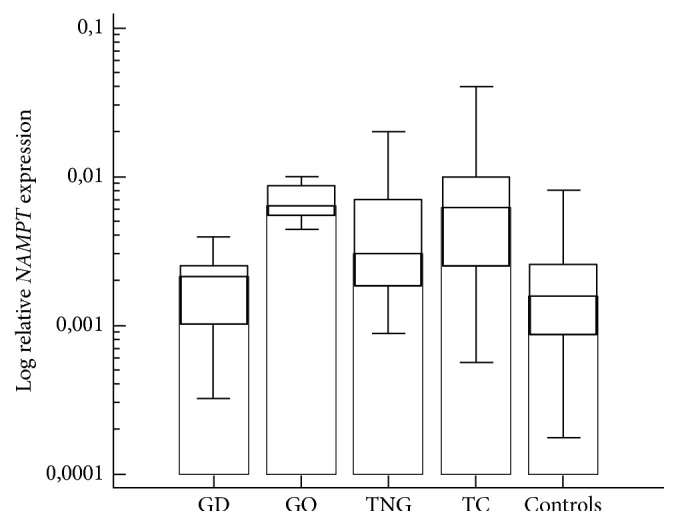
Comparison of log NAMPT expressions between patients with Graves' disease without orbitopathy (GD) and with orbitopathy (GO), toxic nodular goiters (TNG), thyroid cancers (TC), and healthy thyroid tissue samples (controls). The relative quantification with external standard was performed. The* NAMPT* concentration was expressed in relation to concentration of reference/normalization* HPRT* gene. In both cases (*NAMPT* and* HPRT*), standard curves were used to obtain the relative concentration of the target and the reference gene as well as to determine the fit coefficients of the PCR relative standard curve. Results expressed as the target/reference ratio of each sample with the efficiency correction derived from standard curves were logarithmically transformed toward normality.

*NAMPT* expressions were different in the five groups (*p* < 0.000001) (Table 1 and Figure 2). In post hoc analysis, we found the highest* NAMPT* expression in the thyroids of patients with GO and cancers. Also, there was statistically significant* NAMPT* overexpression in patients with TNG. Relatively low* NAMPT* expression was found in GD patients and in the healthy control group.

In one-way ANCOVA, we confirmed that* NAMPT *expression differs between subgroups and that it is not influenced by age, BMI, or sex of patients (*F*(4,142) = 16.252; *p* < 0.001).

## 4. Discussion

To the best of our knowledge, our study is the first evaluating* NAMPT* thyroid gland expression in GD and TNG. We have recently observed* NAMPT* leukocyte overexpression in patients with GO [6]. We aimed to determine if increased* NAMPT* expression might be also found in thyroid gland in Graves' patients. Interestingly, we found significant* NAMPT* overexpression in the thyroid glands of patients with GO, as well as in thyroid cancers. What is more,* NAMPT* expression in Graves' patients without orbitopathy was similar to its level in healthy tissues.

Pathogenesis of Graves' orbitopathy is undetermined, which leads to limited therapeutic options [12, 13]. Numerous cytokines and inflammatory mediators have been identified in orbital tissue and serum of patients with GO [14]. They are potentially involved in the pathogenesis of GO. Increased circulating visfatin serum levels and its overexpression in synovial fibroblasts were found in rheumatoid arthritis (RA) [15, 16]. Observed correlation of NAMPT upregulation with disease severity suggested that visfatin might be a biomarker of RA [17–19]. Enhancement of* NAMPT* expression was also observed in inflammatory bowel diseases as well as in psoriasis [20, 21]. Given these observations, it seems possible that NAMPT/visfatin/PBEF can contribute to ongoing inflammation in autoimmune diseases. NAMPT/visfatin/PBEF displays proinflammatory properties including stimulation of secretion of cytokines such as IL-6, TNF-*α*, and IL-1*β*. NAMPT/visfatin/PBEF also promotes the maturation of both T- and B-lymphocytes. Furthermore, it upregulates costimulatory molecules on monocytes and activates T cells [22]. Taking into consideration the fact that the above-mentioned cytokines are involved in the pathogenesis of Graves' orbitopathy, we could suggest that NAMPT/visfatin/PBEF plays a role in enhancing inflammation in Graves' orbitopathy [23, 24].

We have also found* NAMPT* overexpression in hyperfunctioning thyroid nodules in toxic nodular goiters as compared to Graves' disease without orbitopathy and to healthy controls. However, relative* NAMPT* expression in toxic nodular goiters was still lower than that in thyroid glands derived from patients with Graves' orbitopathy and thyroid cancers. Activating somatic mutations of TSH receptor genes are suggested to be involved in the pathogenesis of TNG [25]. Since these alterations have not always been found in hyperfunctioning nodules, the other mechanisms must play a role, and surely the pathogenesis of toxic nodular goiters has not been fully understood yet. Taking into consideration the crucial role of NAMPT in regulation of cell proliferation, we would explain its overexpression in thyroid nodules. In other words, increased* NAMPT* expression in TNG might be associated with proliferative activity of benign hypersecreting tumor cells. However, in our previous study, we found similar NAMPT expressions in nontoxic nodules and healthy thyroids [6]. Despite the fact that NAMPT is involved in maintaining cell energy balance, its overexpression in hyperfunctioning Graves' thyroids has not been observed. It might result from previous therapy with antithyroid medications to restore euthyroidism before surgical procedure.

Finally, our results might be interpreted in the broader context of a link between autoimmunity and obesity or cancerogenesis [26, 27]. Nowadays, our fundamental understanding of adipose tissue as a regulator of metabolic functions has changed. In view of the studies exploring the role of adipocytokines in immune response and inflammation, fat tissue has emerged as an important factor involved in the pathogenesis of autoimmune diseases. It has been observed that obesity is associated with a higher risk and severity of many autoimmune diseases, for example, systemic lupus erythematosus, IBD, RA, multiple sclerosis, psoriasis, and psoriatic arthritis [28–34]. Recently, higher prevalence of overweight was noticed among patients with GO [35]. Furthermore, intraocular inflammatory cascades resulting in differentiation of preadipocytes and fibroadipose tissue are responsible for the signs and symptoms in GO patients [36]. Enhancement of adiponectin and leptin genes has been observed in orbital preadipocytes [37]. It would be interesting to analyze* NAMPT* expression in retrobulbar fat tissue of GO patients.

We have shown that* NAMPT* overexpression in patients with Graves' orbitopathy and thyroid cancers was at a similar level. In a previous study, we also observed that* NAMPT* expression in thyroid cancers is positively correlated with advanced tumor stage and lymph node involvement [6]. Some authors observed that patients with Graves' disease had larger differentiated thyroid cancers with local relapse many years after thyroidectomy [38–40]. They hypothesized that antiapoptotic interleukins (e.g., IL-4 and IL-10) could contribute to this process. Despite the fact that there are no data reporting that Graves' patients who have eye changes are at higher risk of thyroid cancers, we could speculate that, in the latter group of patients, NAMPT/visfatin/PBEF could be also partially responsible for worse prognosis of thyroid cancers.

## 5. Conclusions

We found* NAMPT *overexpression in thyroid glands of patients with Graves' orbitopathy and thyroid cancers. Reported alteration of* NAMPT* expressions might suggest its involvement in thyroid pathologies. Observed* NAMPT* overexpression in patients with GO and its relatively low levels in thyroids of patients with GD without eye changes do not confirm causal relationship between NAMPT level and orbitopathy, but this might suggest NAMPT involvement in thyroid pathology. Future immunohistochemical analysis should define cellular origin of increased NAMPT expression and its potential association with lymphocytic infiltration. Further experimental studies are needed to explain our findings.

## Figures and Tables

**Table 1:** Characteristics of study groups and results of qPCR reactions.

	GD (*n* = 21)	GO (*n* = 20)	TNG (*n* = 30)	Controls (*n* = 39)	Thyroid cancers (*n* = 40)	*p*
Age [years]	43^a^ (38–55.25)	39^bcd^ (30.5–47)	51.5^c^ (44–59)	54^d^ (44.25–56.75)	56.5^ab^ (40–65)	**0.0029**
Sex [F, females; M, males]	F 19 (90%) M 2 (10%)	F 19 (95%) M 1 (5%)	F 24 (85.7%) M 4 (14.3%)	F 30 (77%) M 9 (23%)	F 24 (60%) M 16 (40%)	**0.001**
BMI [kg/m^2^]	23 (21.9–25.1)	22.5 (20.7–24.6)	23.45 (21.2–26.3)	23.3 (20.9–26.8)	25.16 (21.2–27.5)	0.7569
Cp HPRT	26.08 (25–27.26)	25.78 (25–26.6)	25.25 (24.60–26.36)	24.56 (24.11–25.99)	26.57 (25.57–29.68)	—
Cp NAMPT	27.65 (26.95–29.4)	25.2 (25.27–27.23)	26.94 (26.21–27.61)	24.40 (23.34–26.22)	28.36 (26.8–30.78)	—
Cr-relative NAMPT expression	0.002^abc^ (0.001–0.0025)	0.006^ade^ (0.0055–0.0086)	0.003^bdf^ (0.0019–0.007)	0.006^cg^ (0.0025–0.01)	0.0016^efg^ (0.00087–0.0026)	<0.000001

Data are expressed in medians and interquartile ranges (IQR) provided in brackets; values followed by the same letters differed significantly. GD: Graves' disease; GO: Graves' orbitopathy; TNG: toxic nodular goiter; Cp: crossing point; Cr: concentration ratio.
